# OCT Meets micro-CT: A Subject-Specific Correlative Multimodal Imaging Workflow for Early Chick Heart Development Modeling

**DOI:** 10.3390/jcdd9110379

**Published:** 2022-11-03

**Authors:** Nina Kraus, Fabian Placzek, Brian Metscher

**Affiliations:** 1 Department of Evolutionary Biology, University of Vienna, 1030 Vienna, Austria; 2 Center for Medical Physics and Biomedical Engineering, Medical University of Vienna, 1090 Vienna, Austria

**Keywords:** heart development, optical coherence tomography, micro-CT, correlative imaging, multimodal imaging, chick embryo, heart morphogenesis

## Abstract

Structural and Doppler velocity data collected from optical coherence tomography have already provided crucial insights into cardiac morphogenesis. X-ray microtomography and other ex vivo methods have elucidated structural details of developing hearts. However, by itself, no single imaging modality can provide comprehensive information allowing to fully decipher the inner workings of an entire developing organ. Hence, we introduce a specimen-specific correlative multimodal imaging workflow combining OCT and micro-CT imaging which is applicable for modeling of early chick heart development—a valuable model organism in cardiovascular development research. The image acquisition and processing employ common reagents, lab-based micro-CT imaging, and software that is free for academic use. Our goal is to provide a step-by-step guide on how to implement this workflow and to demonstrate why those two modalities together have the potential to provide new insight into normal cardiac development and heart malformations leading to congenital heart disease.

## 1. Introduction

As developmental processes are highly conserved among amniotes, the chick heart represents an exemplary model for heart morphogenesis in human heart development, particularly because the development of avian embryos inside an egg allows for easy access for cell and tissue manipulations, longitudinal follow up of manipulations, and in vivo imaging [1]. Additionally, just as in humans, the embryonic avian heart undergoes extensive morphological changes during early cardiogenesis: it starts out as a single-chambered tube; through extensive growth and remodeling, it develops into a four-chambered pump with valve leaflets, ventricular trabeculae and double parallel blood flow which, despite some differences, resembles the mammalian and thus, human heart [2]. Most importantly, cardiac defects in human babies can be replicated in avian embryos, allowing a unique developmental window into human congenital heart disease (CHD; [3,4,5,6]. All of this makes the chick model attractive for researchers aiming to understand the mechanistic connection between altered blood flow patterns and congenital heart defects—the most prevalent and often lethal type of birth defect [7]. Attempts at elucidating the role of blood flow in heart development and CHD formation often make use of computational modeling to quantify blood flow velocities and flow stresses.

Subject-specific computational fluid dynamic (CFD) models based on structural and Doppler velocity data collected from optical coherence tomography (OCT) are currently the gold standard in modeling of early cardiac development [8]. This is because OCT allows for non-invasive, label-free, and cross-sectional (B-scan) imaging of tissues in vivo with micrometer-scale spatial resolution [9,10]. So far, OCT is routinely used as a diagnostic tool in ophthalmology and as a fast volumetric guidance during ophthalmic surgery [11], but it is also gaining interest in endoscopy [12], multimodal approaches combining complementary imaging methods to provide deeper understanding of diseases [13], and of signal origin and imaging outcome in diagnosis [14]. Furthermore, functional extensions to OCT can provide additional layers of information to the morphological data: e.g., polarization sensitive OCT [15], Doppler-OCT, or OCT-angiography [16]. Thus, OCT is capable of simultaneous sequence acquisition of structural cardiac images and of blood flow data [8,17]. True subject-specific CFD models are of particular importance when studying treatments or interventions that alter blood flow, especially when they can lead to different blood flow conditions from embryo to embryo [8].

No single imaging modality can give comprehensive information and single-handedly decipher the inner workings of an entire developing organ [18]. Although OCT gives highly resolved in vivo insight into cross-sectional micro structures, the actual dimensions, especially in the axial direction, remain difficult to determine: OCT gives the optical path length in the axial direction. To access the geometrical length in depth, the refractive indices are essential. Usually, an average for the present biological tissue is assumed [19], since refractive index measurements of even homogenous tissue is complex [20]. Therefore, there is a lack of geometrical information, especially for CFD model validation. Furthermore, the OCT imaging depth depends on the optical scattering properties of the investigated tissue and relies on natural tissue contrast for image construction, which limits imaging to the endogenous contrast [21]. Normally, penetration depths range between 1–2 mm in depth, hence it is limited to chick embryonic stages before 3½–4 days of gestation (around Hamburger-Hamilton stage (HH)24; [22]) (out of 21 days of incubation): after 4 days, the embryo starts sinking into the yolk. However, combining different imaging techniques in a multimodal workflow allows overcoming potential shortcomings by integrating the best features of the combined techniques. This provides complementary information about structure, function, dynamics, and molecular composition of the sample [18]. OCT is ideal for correlative multimodal imaging with other modalities as it preserves the tissue, and is non-invasive and label-free. Therefore, sample preparation for OCT imaging does not interfere with any treatments for subsequent imaging.

An especially suitable complementary imaging modality for OCT imaging in early cardiac modeling is ex vivo X-ray microtomography (XRM or micro-CT), as it allows for subject-specific acquisition of genuine and isotropic 3D image volumes at micron resolution [23]. Isotropic refers to equilateral voxels (volumetric pixel) used in a volume data set, where the spatial resolution in the transaxial plane (X-Y plane) and that in the longitudinal direction (Z direction) are equivalent, and spatial resolution is identical in all three spatial directions. This by itself is a great advantage over all anisotropic imaging methods (such as OCT, or reconstruction from serial sections), as this allows one to directly assess the 3D spatial distribution of the cardiac tissue (sub-)structures, such as myocardial fiber orientation, or localization of “tethers” (the ultra-fine connections observed between the endocardium and myocardium), and other morphological features associated with cardiomyopathy [24,25]. Such details in the cardiac microstructures can affect action potential propagation depending on the precise orientation of the cardiomyocytes [26], as well as the mechanical tissue properties and concomitant blood-tissue interactions. Here, the combination of OCT and micro-CT (together with soft tissue mechanics and custom experimental tools [27]) can provide important subject-specific information on how blood flow interacts with the developing cardiac tissue. This is essential for an integrated biomechanical understanding of embryonic heart development [28].

As micro-CT is inherently volumetric, automatically aligned, and size-calibrated, it can also provide a basis for different kinds of quantitative analysis of heart morphogenesis, such as volumetry, morphometry, geometric morphometrics, and densitometry [24,29]. Several studies qualitatively analyzed developmental malformations based on visual inspection and comparison to normal development [29,30,31,32,33]. This makes the addition of micro-CT imaging particularly valuable for studies aiming to understand and phenotype how altered hemodynamic loads caused by certain treatments or interventions can lead to congenital heart defects: OCT imaging can inform about subject-specific changes to hemodynamics and overall heart geometry, while micro-CT can provide subsequent subject-specific information needed to quantitatively describe (micro-)structural alterations to the heart [21,29].

Micro-CT was initially used mainly for mineralized animal tissues [34,35], but the introduction of X-ray dense contrast agents enabled micro-CT imaging of soft (non-mineralized) tissues with high contrast as well [36,37]. This allows the visualization and quantitative description of the structure and microstructure of healthy and diseased cardiovascular tissues during development and in adults using micro-CT. General contrast stains have been in use for over a decade now, and many of them already allowed for valuable insights into heart tissue substructures, such as myocardial fiber direction, or localization of collagen and elastin [38,39,40,41,42]; see [24] for an extensive review on different contrast agents already used in heart imaging and their characteristics. For further microscopic analyses, the sample preparation methods and the non-destructive nature of micro-CT allow for subsequent investigation by histology, scanning electron microscopy, immunohistochemistry, and immunofluorescence [43,44]. However, micro-CT imaging itself is moving in the direction of 3D histology with the development of new tissue-specific X-ray contrast enhancement protocols [45,46,47,48,49] and protocols showing that micro-CT can even be utilized for imaging of molecular signals [50,51]. These new methods will increase the potential of X-ray microscopy for 3D histological investigation of tissues and organisms even further.

**Figure 1:**
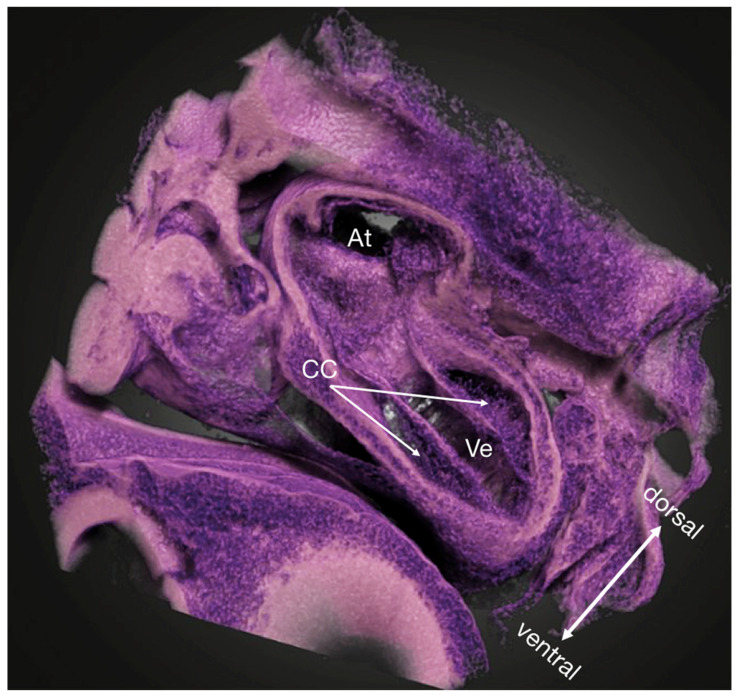
Micro-CT volume rendering of a PbOAc stained HH18 chicken embryo showing a near-histological 3D view into the ventricle and the atrium (arbitrary cutaway). The cardiac cushions are in the process of being populated by cells. Owing to the high specificity of the PbOAc staining, the lookup table and the transfer function could be manipulated as to resemble the classic Hematoxylin/Eosin-staining used in histology to locate nuclei more easily. At atria, CC cardiac cushions, Ve ventricle.

The present study introduces a detailed workflow for specimen-specific correlation of dynamic in vivo OCT imaging of the developing chick heart with high-detail 3D micro-CT data. Our workflow allows for overview and dynamic imaging of the whole heart over the cardiac cycle and can provide 3D virtual histology information in intact fixed specimens (Figure 1). This multimodal approach has the potential to provide new and valuable insights into cardiac morphogenesis by not only overcoming the limitations of isolated standard imaging approaches, but also by providing unique and complementary information only achievable through the correlation of these data. 

As proof of concept, we acquired OCT B-scans of live chicken embryos at HH12-14 in ovo before subsequent fixation in end-diastole, staining in PbOAc, a nuclei specific X-ray dense contrast enhancement [49], micro-CT data acquisition with a commercial laboratory system, and co-registration of the data sets using software that is free to use for academic research. Our goal is to provide an extensive guide facilitating the implementation of this workflow for other research groups and to discuss potential use and the advantages this multimodal approach provides over using a single imaging modality.

## 2. Materials and Methods

### 2.1. Embryo Preparation

Fertilized chicken eggs were incubated blunt-end up at 39.5 °C and 80% humidity for 45–53 h (HH12-14; mid-looping of the heart tube), as is typically done when incubating only up to early developmental stages [22]. Embryos were then removed from the incubator, staged according to Hamburger and Hamilton [22], and prepared for subsequent OCT imaging. The eggs were opened up at the blunt-end by creating a small hole in the shell using a scalpel; tweezers were then used to remove a small area of the shell and the inner shell membrane to access the embryo. Embryos that bled upon membrane removal or that had obvious structural defects were not used for subsequent imaging. All imaging steps were carried out at ambient room temperature and humidity as only a few B-scan positions were imaged per embryo, keeping data acquisition time short. However, for longer imaging time and for accurate assessment of blood flow data, temperature and humidity control during the imaging procedure is highly recommended.

### 2.2. Optical Coherence Tomography

**Figure 2:**
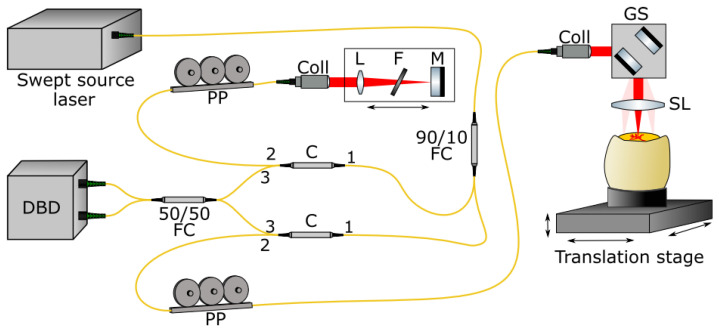
In-house-developed optical setup for performing SS-OCT in a fiber based, Mach-Zehnder configuration. The swept source laser is connected to the interferometer via a 90/10 fiber coupler (FC) transmitting 10% of the optical power to the reference arm via a circulator (C). The collimator (Coll) collimates the laser light, and a lens (L), filter wheel (F), and mirror (M) are mounted on a linear translation stage to match the arm lengths. 90% of the optical power is transmitted to the sample arm in which scanning is performed with galvanometric scanners (GS). The scan lens (SL) focuses the laser light onto the embryo, which is mounted on a three-axis translation stage. Reference and sample arm signals are combined in a 50/50 fiber coupler and detected in a dual balanced detector (DBD). The DBD is connected to the computer for data acquisition as well as driving the swept source laser and the GS. The polarization paddles (PP) provide adjustment capabilities for the polarization state of the laser light.

A custom made, fiber-based OCT system in a laser scanning microscope setup was used in this study. It incorporated an akinetic swept source laser (Insight Photonic Solutions) centered at 1304 nm, providing a bandwidth of 90 nm (rectangular spectrum), leading to an axial resolution of 12µm in air. The sweep frequency was approximately 173 kHz. The scan lens (f = 40 mm; Figure 2) provided a measured lateral resolution of 17.2 µm. The field of view (FOV) can be set to cover 5.5 × 5.5 mm or 2.7 × 2.7 mm with 1000 × 1000 sampling points, exceeding the Nyquist requirement. It is possible to acquire a full three dimensional image stack, or to acquire a cross-section at a specific position (B-scan only), so that cross-sections over time could be acquired from the same position. The pixel size in depth after post-processing was 7.6 µm in air. Assuming a refractive index in tissue of 1.33, the pixel size inside tissue was reduced to 5.7 µm. With an output power of approximately 18 mW on the sample, a sensitivity of 110 dB was measured.

The egg was placed in a tea light holder (IKEA) which can accommodate the pointy end of the egg. The chosen size of the holder was important to not reduce the flexibility of the OCT system in translating the egg in all three directions due to space restrictions. Three small balls of UHU Patafix adhesive putty were put on the walls of the tea light holder to adjust the holder to different sized eggs and to prevent the eggs from moving or tilting. The adhesive putty also allowed us to mount the egg with a slight angle for an improved OCT signal acquisition: this way, strong yolk/egg white surface reflection could be reduced or even removed from the region of interest (ROI). The mounted embryo was placed on a three-axis translation stage to position the embryo precisely to the desired ROI. The live preview of cross-sectional B-scans was used to select an imaging position. After the acquisition of the OCT data (acquisition time < 8 s), raw data is stored on the computer. An in-house developed post-processing pipeline (LabView) was used to perform the Fourier transformation, numerical dispersion compensation and signal optimization of the raw OCT data. Using Fiji, the brightness and contrast was adjusted and a golden lookup table was applied.

### 2.3. Subsequent micro-CT Imaging

**Figure 3:**
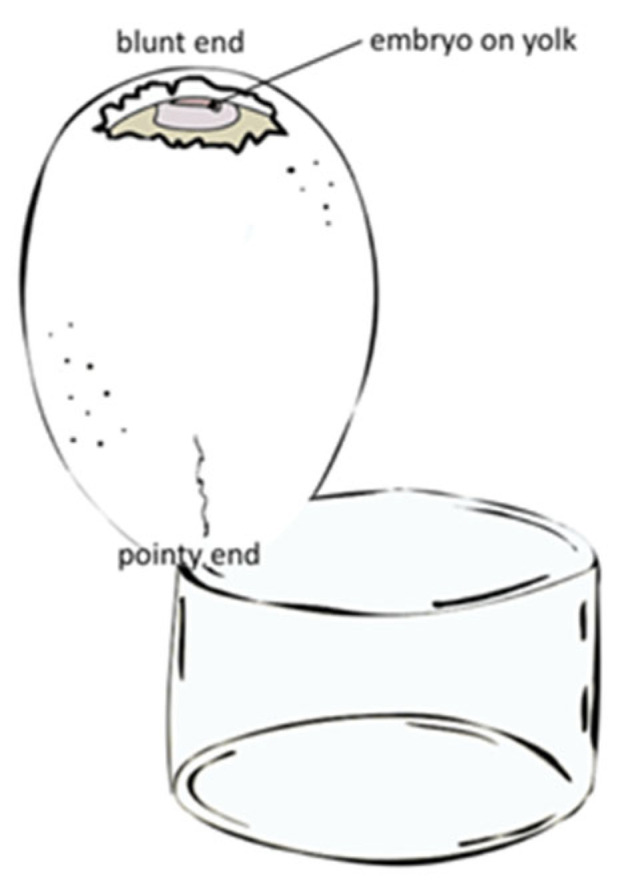
Schematic drawing of embryo preparation and egg orientation required for micro-CT imaging subsequent to OCT image acquisition.

Embryos used for OCT data collection were immediately prepared and fixed in end-diastole (fully relaxed state) for micro-CT imaging. For this, the eggs were carefully cracked into an embryo dish at the pointy end, so that the yolk does not break and the embryo—initially situated on the blunt-end of the egg—now lies on top of the yolk in the dish (Figure 3). Then, the embryo was carefully lifted out of the yolk using a homemade perforated embryo spoon [52] leaving behind as much yolk as possible (as to not dilute the subsequent treatment), while at the same time keeping the blood vessels intact. Once on the spoon, a small hole was made into the embryonic membranes over the ventricle using forceps-scissors, so the treatment reaches the heart more readily, all while making sure the embryo’s heart is still beating.

For fixing the heart in end-diastole, a few drops of 5 mM verapamil/EGTA (0.6 M KCl, 10 mM verapamil, 50 mM EGTA in chick Ringer’s solution) were put directly on top of the ventricle using a sterile transfer pipette (see [5]). For fixing the heart in end-systole (fully contracted), a few drops of 2M NaCl (see [53]) were applied to the ventricle in the same fashion. Once the heart stopped beating, the embryo was cut out of the surrounding blood vessels and embryonic membranes, transferred into an embryo dish containing chick Ringer’s solution to clean it from yolk, and dissected out of residual embryonic membranes with the help of a stereomicroscope. The embryos were then fixed in 4F1G (10% formalin, 1% glutaraldehyde in PBS) to reduce shrinkage. 

After 3 h to overnight of fixation in 4F1G, embryos were given a quick wash in ddH_2_O (>5 min) followed by two 15 min long washes in ddH_2_O. Then, the embryos were contrast-enhanced for Micro-CT imaging using lead(II) acetate trihydrate (PbOAc; CAS 6080-56-4; Sigma-Aldrich 467863) dissolved in distilled water at 2% *w*/*v* [49]. Whole embryos were stained overnight; for hearts dissected out of the embryo at stages up to HH18, 4 h of staining sufficed. Subsequent to staining, specimens were washed again with a quick wash in ddH_2_O (>5 min), followed by two 15-min washes in ddH_2_O. 

After staining, specimens were mounted in polypropylene pipette tips or small polyethylene tubes in 1% low-melting temperature agarose in water [51] for scanning. All specimens used in this study were scanned using a commercial laboratory system, the Xradia MicroXCT microtomography imaging system (Zeiss X-ray Microscopy) with a tungsten X-ray source operated at 40–60 kV and 4 W. Individual projection images were taken every 0.20° over a half-rotation (182–184°) scan, with 40 s exposure times. The tomographic sections were reconstructed with voxel sizes of 0.89–3.99 µm using the XMReconstructor software supplied with the system. The tomograms were reconstructed with the XMReconstructor software supplied with the scanner, and reconstructed tomography volumes were saved in *.txm format.

Obtained images of specimens were examined and described using the Xradia XM3DViewer software, and the free software Dragonfly 3.6 (http://www.theobjects.com/dragonfly, accessed on 31 October 2022) and Fiji (ImageJ; http://www.fiji.sc, accessed on 31 October 2022). 

### 2.4. Additional micro-CT Imaging

To further demonstrate near-histological image quality of micro-CT data over more developmental stages and to demonstrate a more commonly used staining protocol, scanning data of additional specimens was collected. For this, specimens between the stages of HH13-HH21 were used.

For micro-CT imaging only, eggs were incubated as described above, but lying on their side instead of blunt-end up. This way, the eggs could be cracked into an embryo dish on the side, reducing the risk of breaking the yolk. Subsequent embryo preparation and fixation in end-systole and end-diastole was done as described above. This data was also used to see whether fixation at certain points in the heart cycle actually represent reported cardiac-cycle related changes in the cross-sectional shape of the primary ventricular tube. The opened endocardial tube during diastole has an elliptic cross-section, while the collapsed endocardial tube during systole is slit-shaped as a consequence of an uneven distribution of cardiac jelly, which facilitates complete occlusion of the endocardial lumen at end-systole [54,55]. 

The fixed specimens were stained using either the lead(II) acetate trihydrate protocol described above or with 0.3% phosphotungstic acid (PTA) in 70% EtOH [56]. For PTA staining, specimens were washed three times in 70% EtOH for about 15 min each and then transferred to the staining solution for 24–48 h, depending on size. Subsequently, specimens were washed again in 70% EtOH for at least 1 h with one change to fresh 70% EtOH during this period. After staining, specimens were mounted and scanned as described above.

## 3. Results

### 3.1. Embryo Preparation

Optimizing embryo preparation for a multimodal workflow is no trivial matter, as compromises need to be made at each step so that the embryo can be used for both modalities. Incubating eggs blunt side up is the easiest and most reliable way to have the embryo develop at the desired spot and be able to open the egg without causing damage. However, this makes opening up the egg for embryo retrieval more difficult, as the egg now cannot be cracked from the side (easiest breaking point). Opening up at the egg at the blunt-end for OCT imaging makes it necessary to crack it from the pointy-end for subsequent treatment for OCT imaging. However, as this is the most robust part of the egg, utmost care and practice are needed as to not break the yolk; this is crucial with very young embryos (at least up to HH16 in our experience), as otherwise, the tiny, transparent embryo gets lost in the yolk. The methods described above (Figure 3) proved to be the most reliable to facilitate embryo collection for both imaging modalities.

### 3.2. Image Registration

We explored how well micro-CT stacks and OCT B-scans can be correlated and tried to improve the workflow for consistent results other groups can implement in their research. 

**Figure 4:**
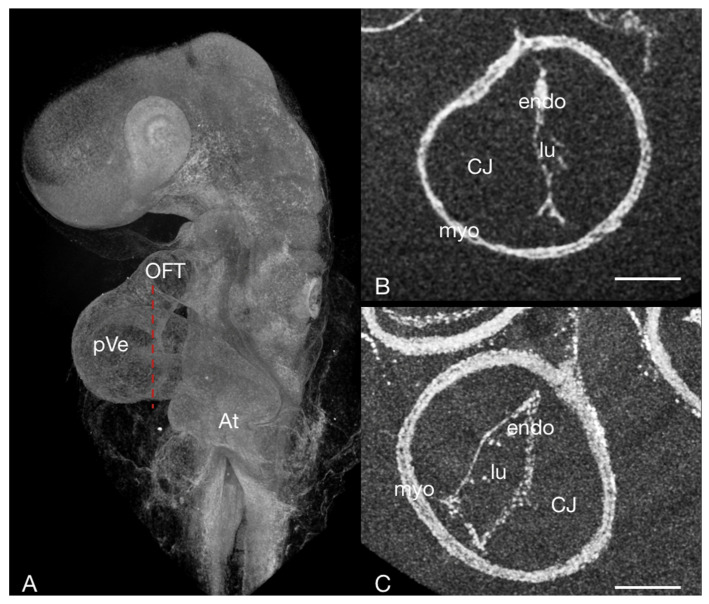
The embryonic heart tube of a HH13 chick stained with PbOAc. (**A**) Overview, micro-CT volume rendering: the red dotted line corresponds to the sectioning plane of (**B**,**C**) Virtual cross-section through the embryonic heart tube in the area of the primitive ventricle fixed at different phases in the heart cycle. (**B**) systole—the lumen is slit-shaped; (**C**) diastole—the lumen appears more oval in shape. At atrium, CJ cardiac jelly, endo endocardium, lu lumen, myo myocardium OFT outflow tract, pVe primitive ventricle; scale bar = 100 µm.

Fixing hearts in end-systole for micro-CT was found to facilitate reliable co-registration. This allowed us to manually choose a B-scan at end-diastole rather than co-registering data for a different, unknown point in the heart-cycle. As the characteristic cardiac-cycle related changes in the cross-sectional shape of the primary ventricular tube reported [54] are also found in specimens fixed at end-diastole (or end-systole), we judged our fixation methods as sufficiently realistic (Figure 4).

It proved to be important that only hearts themselves were co-registered as other positional information (such as the neural tube) could come to lie in different positions in relation to the heart in ovo versus mounted in agarose. Hence, anything but the heart itself was cropped out of the OCT B-scans used. For further processing, *.txm files were converted into *.tiff stacks using a Fiji (ImageJ) plugin [57]. 

For image registration we first opened the required datasets in Dragonfly’s Fusion Mode, adjusting opacity and changing the Lookup Table (LUT) to improve visualization of fused images. To bring the two data sets in close proximity, their centroids were aligned (select one of the datasets in the Data Properties and Settings panel > Align > Centroid with …). Then, data sets were registered manually, which proved to be necessary for adequate results. For this, we first split the view into four equal views (x- y- & z-axis and 3D). After that, the micro-CT data was selected in the Data Properties and Settings panel. Then, by clicking the Move tool in the Move panel (Dynamic refresh on), the Translate and Rotate tools appear in the selected view. Dragging the Translate tool allows to move the selected dataset within the view, the Rotate tool rotates the selected dataset from the pivot position (pivot position can be moved if desired). Those tools were used until adjustments lead to the best co-registration possible. In order to save manual adjustments, the modified datasets have to be exported (e.g., as *.ORSObject or *.tiff). 

**Figure 5:**
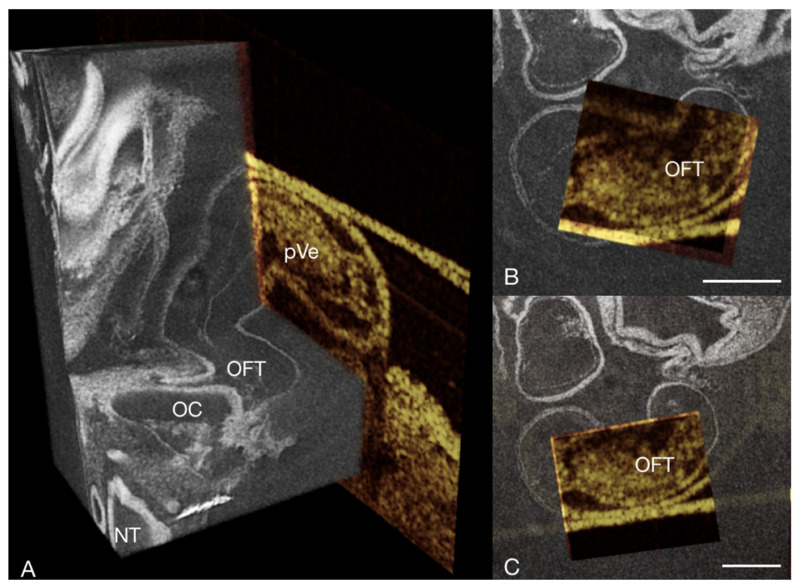
3D micro-CT volume (grayscale LUT) of the heart of a chick embryo stained with PbOAc at HH13 in end-diastole correlated with an OCT B-scan (golden LUT) of the same specimen at the same stage of the heart cycle. (**A**) Virtual cutaway (dorsal) of a micro-CT volume rendering showing the early heart tube correlated with a specimen specific OCT B-scan of the same position of the heart. (**B**,**C**) Rostral view of virtual thick sections in the transverse plane of a micro-CT volume rendering with a window cut out to show the co-registered OCT B-scan of the same specimen. NT neural tube, OC oral cavity, OFT outflow tract, pVe primary ventricle; scale bar = 100 µm.

**Figure 6:**
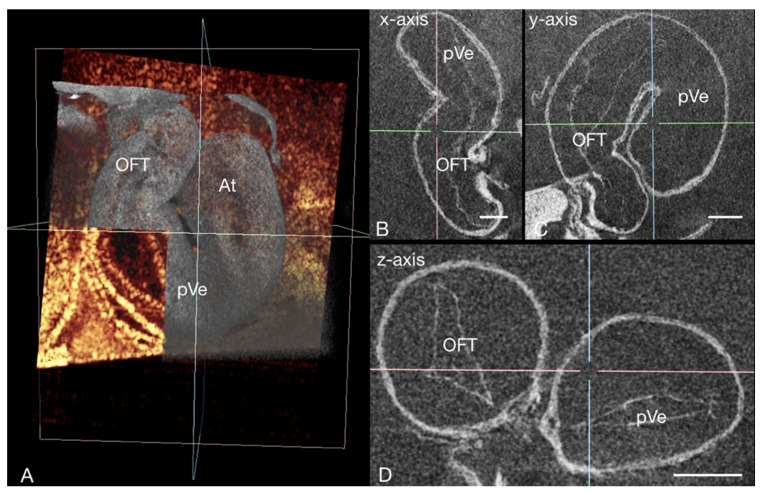
3D micro-CT data (grayscale LUT) of the heart of a chick embryo stained with PbOAc at HH13 in end-diastole, correlated with an OCT B-scan (golden LUT) of the same specimen demonstrating how specimen-specific micro-CT image acquisition subsequent to OCT imaging allows a better understanding of the spatial distribution of cardiac (micro-)structures, as the capability of micro-CT to provide 3D virtual slicing of the whole heart allows for an extensive cardiac assessment. (**A**) Virtual cutaway of a micro-CT volume rendering of the early heart tube co-registered with a OCT B-scan of the same specimen providing an overview of the developing chick heart. The axis position (x: blue, y: pink, z: green) in (**A**) corresponds with the axis position in (**B**–**D**), which shows virtual sections through the heart at different cutting planes. At atrium, OC oral cavity, OFT outflow tract, pVe primary ventricle; scale bar = 100 µm.

All registration procedures in this demonstration used only affine transformations. Thus, elastic transformations between the two data sets (such as minor differences in the heart cycle, small differences in size due to shrinkage caused by the contrasting agent for micro-CT, or uncertainty in actual dimensions in OCT scans) were not compensated. However, this did not affect interpretation as data acquisition at end-diastole allowed for detection and correlation of the same structures with affine transformation (Figure 5 and Figure 6).

### 3.3. Other Findings and Considerations

#### 3.3.1. Staining Used

**Figure 7:**
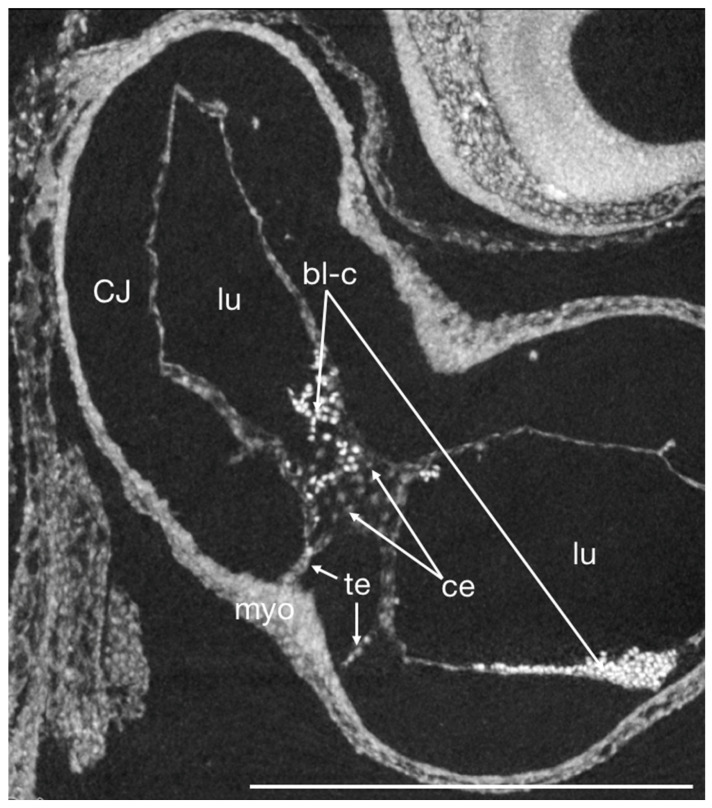
Virtual thick section of a micro-CT scan of a PTA stained HH16 chick embryo heart in sagittal plane showing the OFT and ventricle at early onset of cells migrating into the endocardial cushions (EndMT) and the two-layered myocardium in near histological resolution. The cardiac jelly (CJ) appears empty as the collagens, glycoproteins, and mucopolysaccharides that it consists of do not stain sufficiently with PTA to be detectable at this resolution. Note that blood cells stain heavily with PTA. bl-c blood cells, ce cell nuclei, CJ cardiac jelly, endo endocardium, lu lumen, myo myocardium, te tether. scale bar = 500 µm.

**Figure 8:**
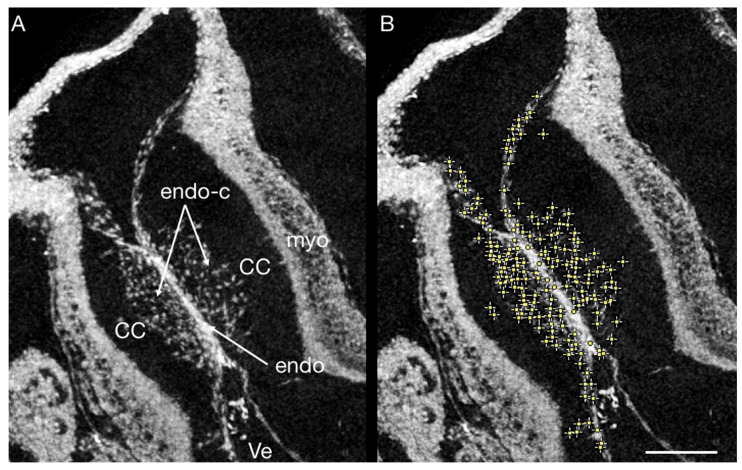
(**A**) Virtual thick sections of a PbOAc stained HH17 chick OFT. The cardiac cushions are invaded by endothelial cells undergoing EndoMT. (**B**) Maxima in grayscale values found in a selected area (cardiac cushions) using Fiji’s (ImageJ) “Find Maxima” tool (yellow dots; here, maxima correspond to nuclei, as PbOAc is nuclei-specific, which means they absorb more X-rays and therefore have higher grayscale value). CC cardiac cushion, endo endothelium; endo-c endothelial cells, myo myocardium, Ve ventricle; scale bar = 100 µm.

We decided to focus on PbOAC staining [49], as it is more specific to nuclei, meaning that more of the staining agent binds to nuclei than to other components of the imaged tissue; blood cells stain especially heavily with PTA (Figure 7). This is valuable in understanding cardiac development as alteration in the number of cells migrating into cardiac cushions during endothelial to mesenchymal transition (EndoMT) have been associated with CHD. The process of EndoMT is responsible for populating the cushions with cells crucial for normal development into functional heart valves [58,59,60,61]. In normal heart development, the number of cells found in the cardiac cushions has been found to increase linearly over development, but when disrupted by hemodynamic interventions, the rate of EndoMT is altered [5,60,62]. The resolution of both OCT [63] and micro-CT (Figure 7) allows for assessing EndoMT via the cell-counting method. While OCT is ideal for longitudinal progression of EndoMT, micro-CT allows more robust positioning of the imaging plane and assessment of the spatial distribution of invading cells. The inherent optical tissue contrast of OCT suffices for cell imaging in the developing cardiac jelly. However, when evaluating the number of invading cells, we recommend PbOAc staining [49]. Other stainings, such as PTA, also allow the visualization of nuclei (Figure 7), but when stained with PbOAc, nuclei absorb more X-rays and therefore have higher grayscale values than other tissues components (see Figure 1). Based on this, we were able to use Fiji’s (ImageJ) “Find Maxima” tool on a virtual thick section of a selected area of the OFT cushions to evaluate the number of invading cells (Figure 8). Cells thus marked can then be enumerated using the cell counting tools in Fiji or other software. 

However, co-registration proved to be independent of whether PbOAc or PTA was used for X-ray contrast enhancement. We are confident that all general contrast agents will provide sufficient contrast for co-registration of the heart tissues. However, some stainings (such as PTA) can cause significant tissue shrinkage, which would lead to less precise co-registration. Whenever possible, we recommend stains that are already less prone to cause shrinkage, such as PbOAc. When using a very tissue-specific contrasting agent, dual energy micro-CT (microDECT; [64]) might be necessary to simultaneously image the histology and the general morphology.

#### 3.3.2. Using micro-CT for Doppler OCT Based CFD Model Validation

Micro-CT data has frequently been used by itself for creating the flow domain of a CFD model (e.g., [60]), as it can be combined with ultrasound methods which do not provide geometric information. Micro-CT also provides a more direct way to create the geometry, as the anisotropic nature of OCT and the motion of the beating heart poses a challenge. However, this of course only provides static information. Hence, methods facilitating the acquisition of the 3D vessel geometry from OCT data have been developed [65,66], allowing for more realistic, dynamic CFD models. Nonetheless, OCT still relies on refractive indices averaged over all tissues to provide geometric information, leading to inaccurate heart lumen diameters that could affect the accuracy of CFD models. Many efforts are made to precisely measure and correct for different refractive indices [67,68,69], but whole organs, like the developing chick heart, are made up of many different tissues, complicating any attempts at acquiring realistic geometries from OCT data.

As micro-CT data is calibrated in geometry, it could, in theory, serve as an excellent tool for validating the geometry obtained from OCT imaging. However, tissue preparation necessary for micro-CT imaging can lead to significant tissue shrinkage, caused by the fixation and staining method used [29], among other things. However, tissue shrinkage can be minimized either by polymer perfusion [70] or by the combination of formaldehyde-fixation, hydrogel-embedding [71], and neutral-buffered iodine infiltration [72]. Hence, careful embryo preparation for micro-CT imaging in combination with our proposed workflow could circumvent the technical challenge of correcting for different refractive indices in the multi-layered heart tube, while still allowing for specimen-specific geometry validation: cross-sectional OCT B-scans at different positions of the heart tube in end-diastole would need to be co-registered with the specimen-specific end-diastole fixed micro-CT data. Then, the lumen surface needs to be assessed for all cross-section images using OCT. In Dragonfly ORS, this is done by segmenting lumen in the virtual slices and B-scans of interest; basic measurements like surface size are then calculated automatically if the image data was loaded with the correct voxel size. Then, the quotient of the lumen surfaces of the same position in an OCT B-scan and a micro-CT scan can be used to validate the dynamic geometry retrieved from OCT imaging to model changes in blood flow over the cardiac cycle even more accurately.

#### 3.3.3. Reproducibility

During OCT imaging of the embryo, the strong signal of the interface between air and egg needs careful handling. Depending on where exactly the embryo lies within the egg, this strong reflection can potentially saturate the detector and create image artifacts overlaying the beating heart. This is particularly problematic if the embryo lies close to the surface or in the middle of the opening, where the surface is more flat than curved, which causes stronger reflections. Besides refractive index matching by adding a few drops of chick Ringer’s, we found that tilting the egg to orient the interface between embryo and yolk/egg white oblique to the incident laser beam helped to reduce strong back reflection artifacts. Thus, the area of strong detected signal from the surface can be properly managed. 

Regarding reproducible B-scan orientation, variations in egg size and embryo positioning require individual changes to the settings of the OCT system with respect to the reference arm length and the z-position of the egg itself. The live preview of the OCT data is helpful for the desired orientation of the embryo. Experience with the mentioned parameters led to a reproducible OCT data acquisition from the chicken embryos.

With micro-CT imaging, the orientation of the specimen during image acquisition does not matter, as the isotropic 3D image volumes can be arbitrarily resliced. However, the times for staining we specified might need to be adjusted depending on sample size.

## 4. Discussion

We have demonstrated a correlative multimodal imaging workflow using OCT and micro-CT imaging for early development heart modeling in the chick. As discussed, both OCT and micro-CT imaging individually have already greatly enhanced our understanding of cardiac morphogenesis and the interaction of hemodynamics with the developing heart, but the integration of both into a multi-modal workflow will allow for new insights impossible to achieve using either method alone.

OCT delivers dynamic in vivo anatomical and morphological information at acquisition speeds of up to multiple volumes per second [73], and also functional information if Doppler OCT is applied to detect axial blood flow velocity at the same spatiotemporal resolution as structural OCT [21]. Micro-CT data provides high-detail isotropic 3D data that is calibrated for both geometry and intensity and that can be acquired over a large number of developmental stages [29]. The combination of these modalities, however, not only overcome the limitations of isolated standard imaging approaches, but also provide unique and complementary information only achievable through the correlation of these data. Integrating those two modalities is particularly valuable when studying treatments or interventions that alter blood flow in order to replicate CHD, especially when those interventions can lead to varying blood flow conditions from embryo to embryo [8].

Even without subsequent analysis, the proposed workflow will allow for a deeper understanding of the downstream effects hemodynamic interventions can have on cardiac morphogenesis, as many visible developmental changes are already known to be associated with CHD. We hope to provide a useful guide on how to implement this workflow and to have demonstrated that a vast amount of open questions about cardiac development can be addressed using this multimodal approach. The potential of this multimodal approach has not yet been fully explored, and the integration of OCT and micro-CT imaging will allow for even further insights into heart development in the future. With the continued advancement of technology, the potential for studying heart morphogenesis will expand even further. While micro-CT imaging is already a well-established technique, efforts for moving toward virtual 3D histology are only just starting to take off. 

With the development of new, tissue-specific contrast agents, even more specific questions aiming to better our understanding of normal cardiac development and the formation of CHD can be addressed with the presented workflow. However, despite expected advancements in contrast agent development, the low molecular sensitivity of micro-CT imposes a limit on its use for analyzing molecular signals. While potential ways of amplifying weak signals are feasible, another way of circumventing this limitation could be the use of micro-CT as a bridge technique. As both OCT and micro-CT are non-destructive, they lend themselves to integrating even further imaging modalities [21,29,74,75]. In such a workflow, micro-CT imaging will provide complete, isotropic 3D data sets enabling easy co-registration and co-localizations of ROIs in OCT data with either histology or immunohistochemistry, allowing for subsequent downstream analysis [29,43]. 

## Data Availability

The original tomographic image data are available on request from the author.
